# Impact of the COVID-19 pandemic on prehospital emergency medical service: a scoping review

**DOI:** 10.3389/fpubh.2025.1543150

**Published:** 2025-03-19

**Authors:** Hannah Richter, Marlieke Schneider, Johanna Eisenberger, Nastaran Jafari, Hannah Haumann, David Häske

**Affiliations:** ^1^Center for Public Health and Health Services Research, University Hospital Tübingen, Tübingen, Germany; ^2^Center for Quality Management in Emergency Medical Services Baden-Wuerttemberg (SQR-BW), Stuttgart, Germany; ^3^Institute for General Medicine and Interprofessional Care, Tübingen University Hospital, Tübingen, Germany

**Keywords:** emergency medical services, emergency medicine, EMS, emergency calls, emergency operations, COVID-19, coronavirus, SARS-CoV-2

## Abstract

**Background:**

The COVID-19 pandemic has had an unprecedented impact on healthcare systems worldwide. Emergency medical services (EMS) frequently served as the sole point of contact for individuals in need of assistance or emergency support. This study aimed to map the impact of the pandemic on emergency calls and EMS operations.

**Methods:**

A systematic literature search was conducted in the electronic databases Pubmed and Web of Science. A hand search supplemented the search. Published articles in English or German dealing with frequencies, diagnoses, and factors influencing emergency calls and EMS use were included. Studies on cardio-pulmonary resuscitation were not included.

**Results:**

The initial search yielded 3,359 articles, of which 3,187 were screened by title/abstracts, and 120 full-text articles were analyzed. Fifty articles were then included. Fourteen articles reported the number of emergency calls, 30 on the number of EMS operations, and six on both outcomes. The articles were mostly published in 2020 (*n* = 18) or 2021 (*n* = 29) and dealt with the situation of EMS during the COVID-19 pandemic in 13 European countries and 11 non-European countries. However, the quantitative data on changes in emergency calls show considerable variation (standard deviation of 31.3% with a mean of 0.0%, minimum: −50.0% to maximum: 121.0%). The quantitative data on changes in EMS operations show a more significant overall decrease (mean: −12.2%, standard deviation: 24.7%, minimum: −72% to maximum: 56%).

**Conclusions:**

The heterogeneity of the studies is considerable; overall, there appears to have been a decline in emergency calls, particularly EMS operations. Clear patterns, e.g., by region, cannot be identified.

**Review protocol registration:**

The review protocol is registered in the Open Science Framework: https://osf.io/8urq9.

## 1 Introduction

The novel coronavirus SARS-CoV-2 (COVID-19) detected at the end of 2019 has had extreme consequences worldwide. On March 11 2020, the World Health Organization declared COVID-19 a global pandemic (1). In many countries, public life was severely restricted after that. Public events were canceled, schools and universities were closed, and “Stay-at-Home Restrictions” and “lockdowns” were implemented (2–4). Where possible, employees were sent to home office to reduce infection rates in workplaces (3).

To date, numerous studies have examined the Impact of COVID-19 on a variety of areas, such as the education system (5, 6), the economy (7, 8), and the healthcare system (9, 10). The COVID-19 pandemic tested healthcare systems worldwide and significantly impacted the functionality and utilization of healthcare and emergency medical services (EMS) (1). Studies almost universally reported declining healthcare utilization for diseases other than COVID-19, and it is noteworthy that this trend was observed for both routine and emergency services (10). Many countries experienced a decrease in patient contacts, but also the planned suspension of elective procedures and a consecutive redistribution of clinical staff to relieve colleagues in intensive care units and COVID-19-associated patient care, which led to a disruption in care (1).

Prehospital emergency medical services (EMS) deserve special attention in the healthcare sector. These EMS providers treat and transport patients for various indications, usually without prior knowledge of the patients. Numerous country-specific studies have documented this phenomenon by examining the impact of the coronavirus disease 2019 (COVID-19) pandemic EMS. However, to the best of our knowledge, no comprehensive overview exists. This review will analyze the impact of the pandemic on the number of emergency calls and EMS operations, and specific diagnoses will break both down.

## 2 Methods

### 2.1 Protocol and registration

Given the considerable heterogeneity of the expected publications, we conducted a scoping review on the frequency of EMS calls and EMS operations during the COVID-19 pandemic. This work adheres to the methodological framework of Arksey and O'Malley and the PRISMA Statement Extension for Scoping Reviews (PRISMA-ScR) (11, 12). The study protocol is registered in the Open Science Framework: https://osf.io/8urq9.

### 2.2 Eligibility criteria

The present study has included published articles in English or German examining the frequency, type of diagnoses, and factors influencing ambulance operations and emergency calls (Table 1). These influencing factors may include demographic characteristics (e.g., age, gender, socioeconomic status), temporal patterns (e.g., seasonal or diurnal variations), geographic influences (e.g., countries, continents), and system-level variables, among others.

**Table 1:** PICOS-Schema.

Patient/population	Patients in the emergency medical service (EMS operations) or seeking help *via* emergency call
Intervention	Influence of the COVID-19 pandemic worldwide
Comparison	Comparison of pandemic phases with no pandemic (pre/post)
Outcome	Frequencies
Studies	Empirical research work

Studies focusing primarily on cardiopulmonary resuscitation (CPR) were excluded from consideration due to their unique clinical and operational considerations, which are the focus of a separate review. The inclusion criteria were chosen to ensure a comprehensive understanding of the broader landscape of EMS interventions and emergency call dynamics. Analyzing the frequency and type of EMS calls and the factors that influence these events aims to identify patterns and potential areas for system improvement. The exclusion of studies dealing with CPR is justified by their highly specialized nature, which often involves different research methods, patient populations, and outcome measures than general EMS interventions.

### 2.3 Search

We conducted a systematic literature search in the electronic databases Pubmed and Web of Science using the following search terms and filters: (“Emergency Medical Services” [Mesh] AND “SARS-CoV-2” [Mesh]) OR (“Emergency Medical Services” [Mesh] AND “COVID-19” [Mesh]) and (“EMS” OR “Emergency Medical Service” OR “ambulance” OR “Prehospital”) AND (“COVID-19” OR “SARS-CoV-2”). A hand search supplemented the search. The searches took place between June and December 2021. The date of the most recent search was September 16, 2021.

### 2.4 Selection of studies

**Figure 1:**
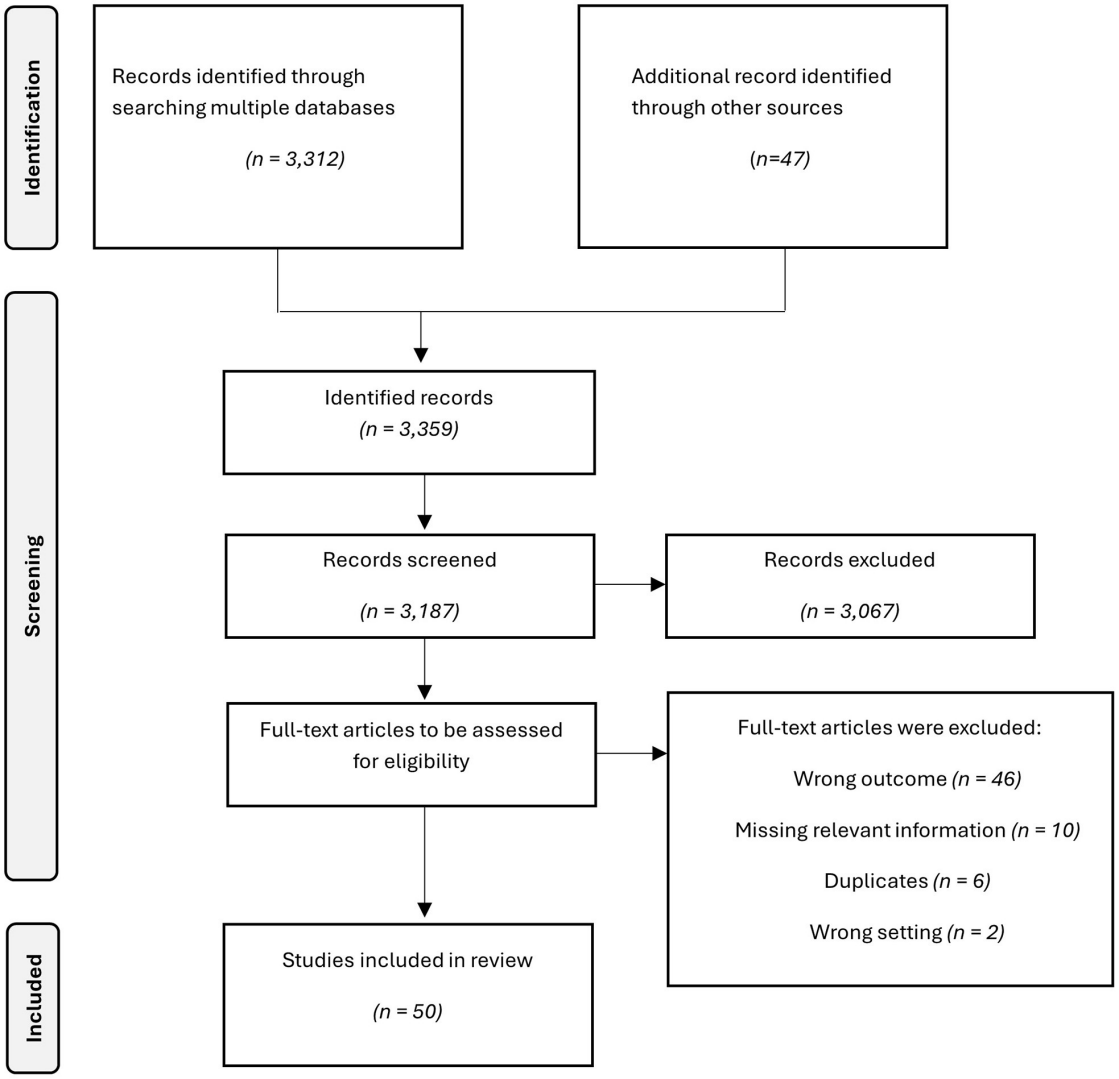
PRISMA flow chart for the study selection process.

The literature search resulted in a total of 3,359 articles. After removing duplicates, 3,187 articles were screened by titles and abstracts. Based on title and abstract, 3,067 were excluded, and 120 full-text articles were retrieved and assessed for eligibility. The full-text screening resulted in the exclusion of 70 studies for the following reasons: 46 reported other outcomes (e.g., out-of-hospital cardiac arrest), relevant information was missing in 10 studies, 6 were duplicates that were not identified in advance because they were published in different languages, 2 reported the impact of the COVID-19 pandemic on another setting like hospitals. There was no full-text for two articles; one was published in another language. Finally, 50 articles were included in this scoping review. Figure 1 shows the complete study selection process in a PRISMA flow chart.

### 2.5 Data charting process

The indexed publications were reviewed independently by two reviewers using Rayyan software to check titles and abstracts [Qatar Computing Research Institute (Data Analytics), Doha, Qatar] (13). Any discrepancies between the reviewers at any stage of the selection process were resolved through discussion or by a third reviewer. The full text of selected citations was reviewed in detail. Reasons for excluding literature in the complete text analysis phase were recorded.

Data extraction was done using a standardized data abstraction table developed for this scoping review. The table included relevant information on study characteristics (year of publication, outcomes, country, and study population), detailed information on study periods, and described changes in EMS calls and operations. All patients included in the studies were considered. Based on an initial literature search and our own experience, additional subgroup analyses from the studies on the following diagnoses were included in the table: cardiac emergencies, respiratory diseases, traumata, mental health conditions, and intoxications. The percentage changes presented in the studies are included in the table. If absolute values were given, percentage changes were calculated. If a percentage change was neither shown nor could be calculated, the only information included in the table was whether an increase, a decrease, or no change was observed. Two reviewers carried out data extraction. Ambiguities and uncertainties were discussed with a third reviewer.

For the figures that show the frequencies of call-outs or emergency calls, the relative frequencies in terms of increase or decrease were presented in bar charts, as well as the mean change in the respective diagnostic groups and the 95% confidence interval in the further figure.

## 3 Results

### 3.1 Characteristics of included studies

**Figure 2:**
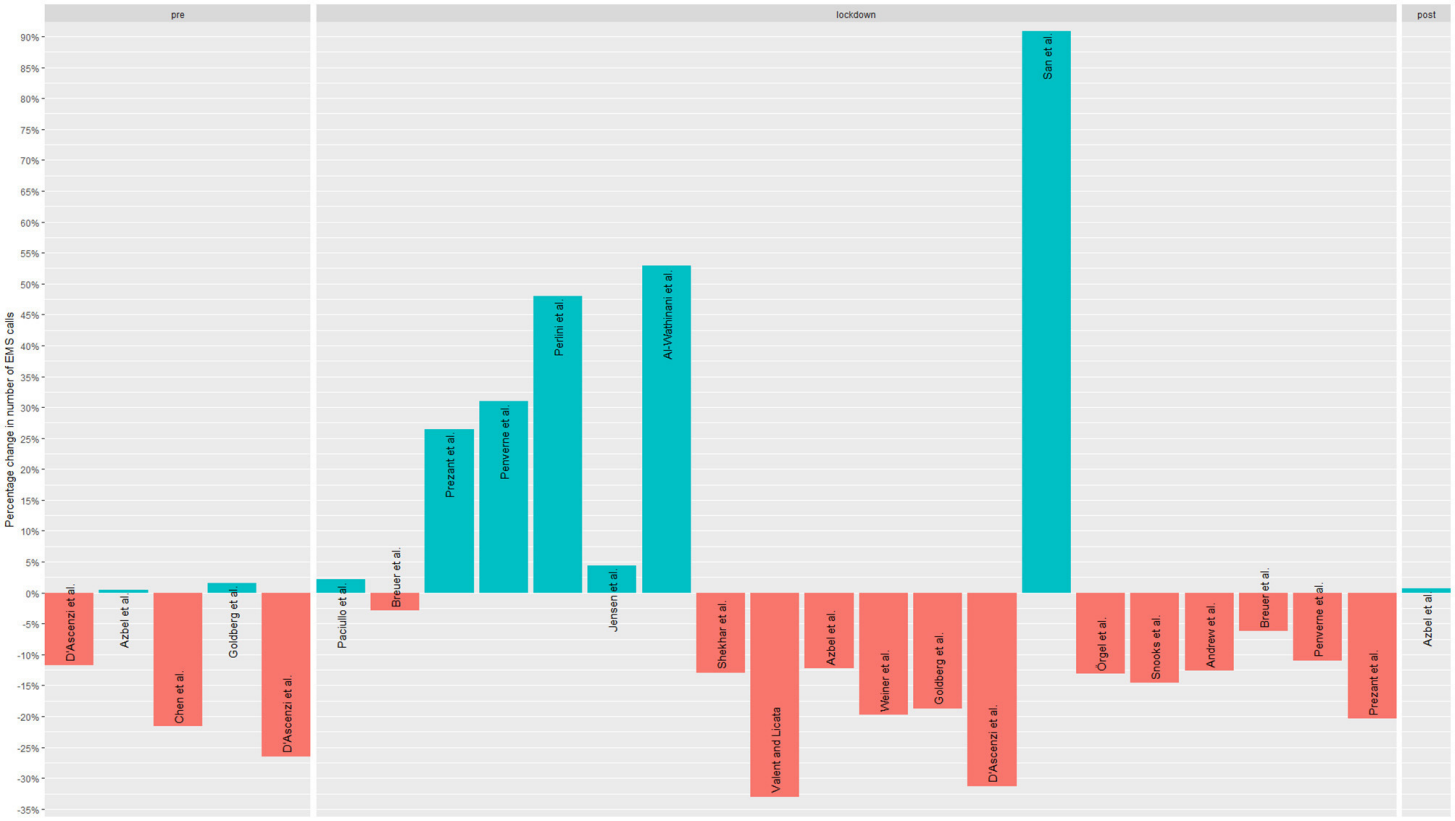
Studies' reported changes regarding EMS calls.

**Figure 3:**
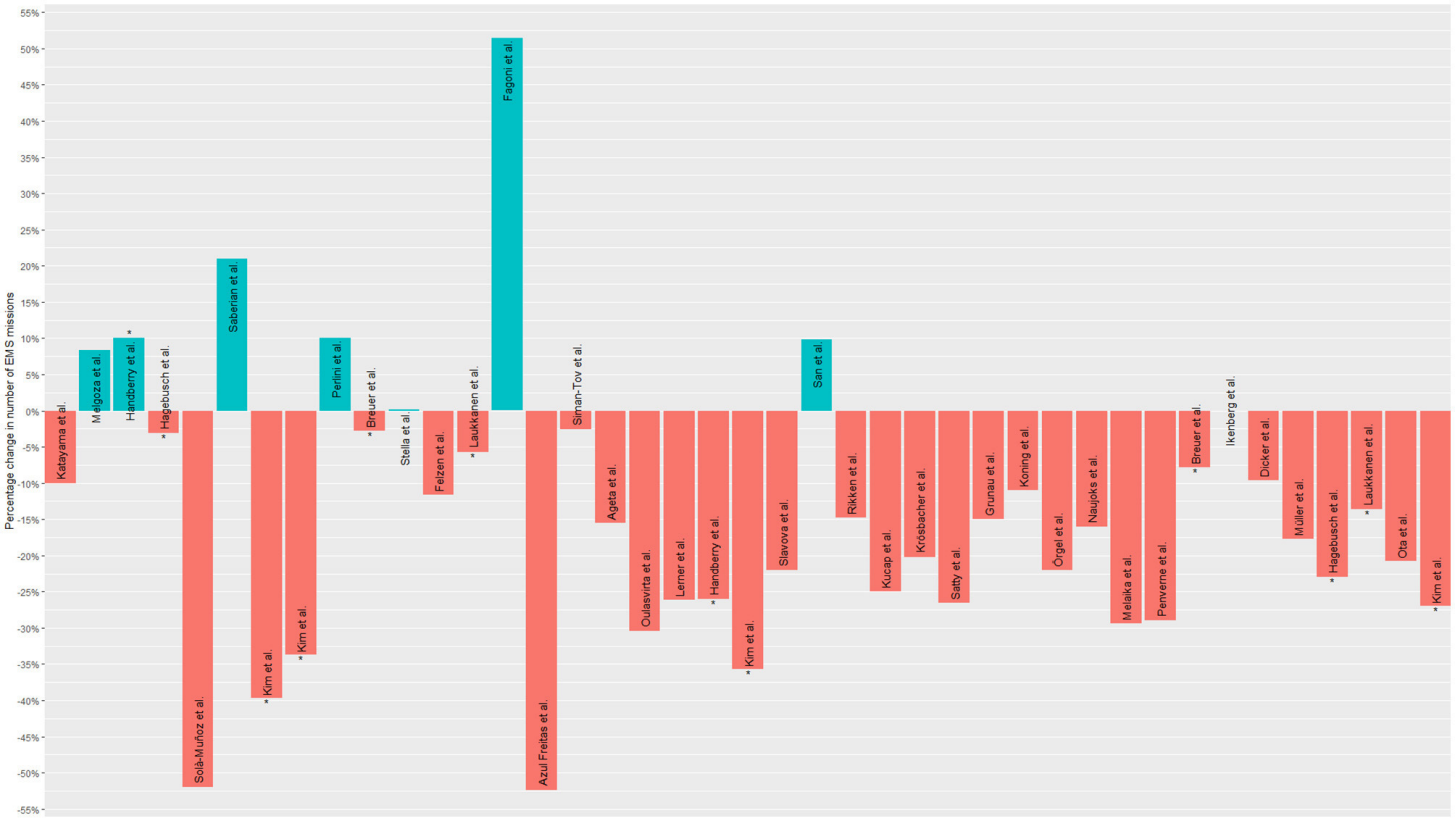
Studies' reported changes regarding EMS operations. In the graph, those studies that appear more than once (because they compared different periods) are marked with an asterisk (^*^).

**Figure 4:**
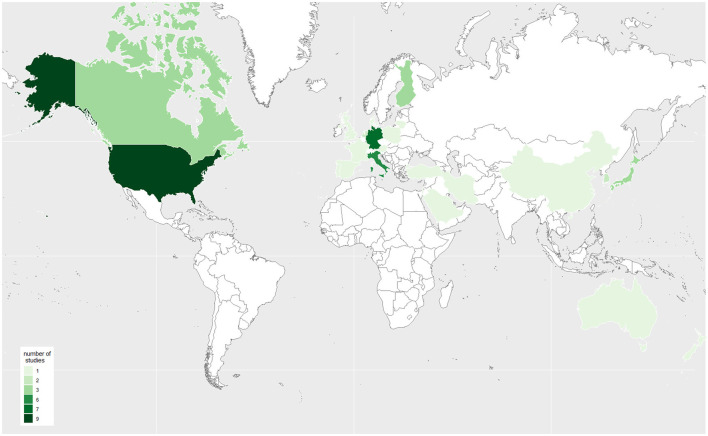
Origin of the included studies (the darker the color the more studies included).

Table 2 shows the study characteristics, including study outcomes, year of publication, country of origin, and study population. Of the 50 articles included, 14 reported the number of EMS calls, 30 reported the number of EMS operations, and six reported both outcomes (Figures 2, 3). The articles were mostly published in 2020 (*n* = 18) or 2021 (*n* = 29) and covered the situation of EMS services during the COVID-19 pandemic in 13 European countries and ten non-European countries. Countries most frequently represented among the included studies were the USA (*n* = 9), Germany (*n* = 7) and Italy (*n* = 6) (Figure 4). Most studies reported the number of EMS calls or operations for all patients. Two articles are limited to specific age groups (14, 15), and eight are limited to specific stroke diagnoses (16–23). In some studies, in addition to analyses of all patients, additional subgroup analyses were conducted (Supplementary material). The study periods varied between the included articles (Supplementary material). Most studies reported the first months of the COVID-19 pandemic, which often included a lockdown. Others reported more extended periods, e.g., about a year. Most articles compared the number of EMS calls or operations with those from the corresponding period in the previous year(s). Others compared to the months before the outbreak of the COVID-19 pandemic or reported variations in the months of the pandemic. Some studies compared several periods.

**Table 2:** Study characteristics.

**Author**	**Year of Publication**	**Outcome**	**Country**	**Population**
Al-Wathinani et al. (24)	2020	EMS calls	Saudi Arabia	All patients
Andrew et al. (25)	2021	EMS calls	Australia	All patients
Azbel et al. (26)	2021	EMS calls	Finland	All patients
Chen et al. (27)	2022	EMS calls	China	All patients
D'Ascenzi et al. (17)	2021	EMS calls	Italy	Patients with cardiac problems
Ferron et al. (28)	2021	EMS calls	Canada	All patients
Goldberg et al. (29)	2021	EMS calls	USA	All patients
Jensen et al. (30)	2021	EMS calls	Denmark	All patients
Paciullo et al. (31)	2021	EMS calls	Italy	All patients (focus: out-of-hospital ACSs, strokes and cardiac arrests)
Prezant et al. (32)	2020	EMS calls	USA	All patients
Shekhar et al. (22)	2020	EMS calls	USA	Patients with cardiovascular events
Snooks et al. (33)	2021	EMS calls	U.K.	All patients
Valent, Licata (34)	2020	EMS calls	Italy	All patients
Weiner et al. (35)	2021	EMS calls	USA	All patients (focus: patients with substance-related issues)
Ageta et al. (36)	2020	EMS operations	Japan	All patients
Azul Freitas et al. (16)	2021	EMS operations	Portugal	STEMI-patients
Dicker et al. (37)	2020	EMS operations	New Zealand	All patients
Fagoni et al. (38)	2021	EMS operations	Italy	All patients
Felzen et al. (39)	2020	EMS operations	Germany	All patients
Grunau et al. (40)	2021	EMS operations	Canada	All patients
Hagebusch et al. (41)	2020	EMS operations	Germany	All patients
Handberry et al. (42)	2021	EMS operations	USA	All patients
Ikenberg et al. (18)	2020	EMS operations	Germany	Patients with Stroke
Katayama et al. (19)	2020	EMS operations	Japan	Patients with acute diseases and traffic accidents
Kim et al. (43)	2020	EMS operations	South Korea	All patients
Kim et al. (20)	2021	EMS operations	South Korea	Patients with acute stroke who received reperfusion therapy
Koning et al. (21)	2021	EMS operations	Netherlands	Patients with Chest pain or out-of-hospital cardiac arrest (OHCA)
Krösbacher et al. (44)	2021	EMS operations	Austria	All patients
Kucap et al. (45)	2020	EMS operations	Poland	All patients
Lane et al. (46)	2021	EMS operations	Canada	All patients
Laukkanen et al. (47)	2021	EMS operations	Finland	All patients
Lerner et al. (48)	2020	EMS operations	USA	All patients
Melaika et al. (49)	2021	EMS operations	Lithuania	Patients with a suspected stroke or transient ischaemic attack
Melgoza et al. (14)	2021	EMS operations	USA	Patients aged 50 and older
Müller et al. (50)	2022	EMS operations	Germany	All patients
Naujoks et al. (51)	2023	EMS operations	Germany	All patients
Ota et al. (52)	2022	EMS operations	Japan	All patients
Oulasvirta et al. (15)	2020	EMS operations	Finland	All patients aged 0–15 years
Rikken et al. (53)	2021	EMS operations	Netherlands	All patients
Satty et al. (54)	2021	EMS operations	USA	All patients
Siman-Tov et al. (55)	2021	EMS operations	Israel	All patients
Slavova et al. (56)	2020	EMS operations	USA	All patients (focus: Patients with opioid intoxication)
Solà-Muñoz et al. (23)	2021	EMS operations	Spain	Polytrauma patients
Stella et al. (57)	2020	EMS operations	Italy	All patients
Breuer et al. (58)	2021	EMS calls	Germany	All patients
		EMS operations		
Örgel et al. (59)	2021	EMS calls	Germany	All patients
		EMS operations		
Penverne et al. (60)	2021	EMS calls	France	All patients
		EMS operations		
Perlini et al. (61)	2020	EMS calls	Italy	All patients
		EMS operations		
Saberian et al. (62)	2020	EMS calls	Iran	All patients
		EMS operations		
San et al. (63)	2021	EMS calls	Turkey	All patients
		EMS operations		

### 3.2 Changes regarding EMS calls

The articles reporting quantitative data on changes in emergency calls demonstrate a mean change of 0.0%. However, considerable variation exists, with a standard deviation of 31.3% (minimum: −50.0% to maximum: 121.0%). Of the 20 articles that reported changes regarding EMS calls, nine reported a decrease (17, 22, 25, 27, 28, 33–35, 59), varying from −4.62% (22) to −33.02% (34) (Supplementary material). They presented the situation in Australia (25), China (27), Italy (17, 34), Canada (28), Germany (59), the USA (22, 35) and U.K. (33). An increase in EMS calls was observed in six studies, varying from +2.14% (31) to +90.9% (63) (Supplementary material). An increase of +347% (62) represented a substantial outlier value. An increase was reported in Saudi Arabia (24), Denmark (30), Italy (31, 61), Iran (62) and Turkey (63). Five studies reported mixed trends in the number of emergency calls in Finland (26), Germany (58), the USA (29, 32), and France (60), depending on the study period.

### 3.3 Diagnosis-specific changes regarding EMS calls

EMS calls on cardiac emergencies, respiratory diseases, trauma, mental health conditions, and intoxication were considered (Supplementary material). Eleven studies examined changes in EMS calls for cardiac emergencies (17, 22, 24, 25, 27–29, 31, 32, 34, 60). Five studies reported increased calls for cardiac emergencies, three reported a decrease, and three had mixed trends. Changes in EMS calls for respiratory diseases were examined in six studies (28, 29, 32, 34, 35, 60). Four articles reported an increase, and two articles reported a decrease. Six studies reported changes for traumata (24, 26, 28, 32, 34, 60), five of which observed a decrease, and one article reported mixed trends depending on a specific diagnosis. Changes in EMS calls for mental health conditions are the subject of three articles (25, 28, 32). Two reported an increase, and one a decrease. Three studies reported changes in intoxication (28, 35, 60); one stated an increase and two a decrease.

### 3.4 Changes regarding EMS operations

**Figure 5:**
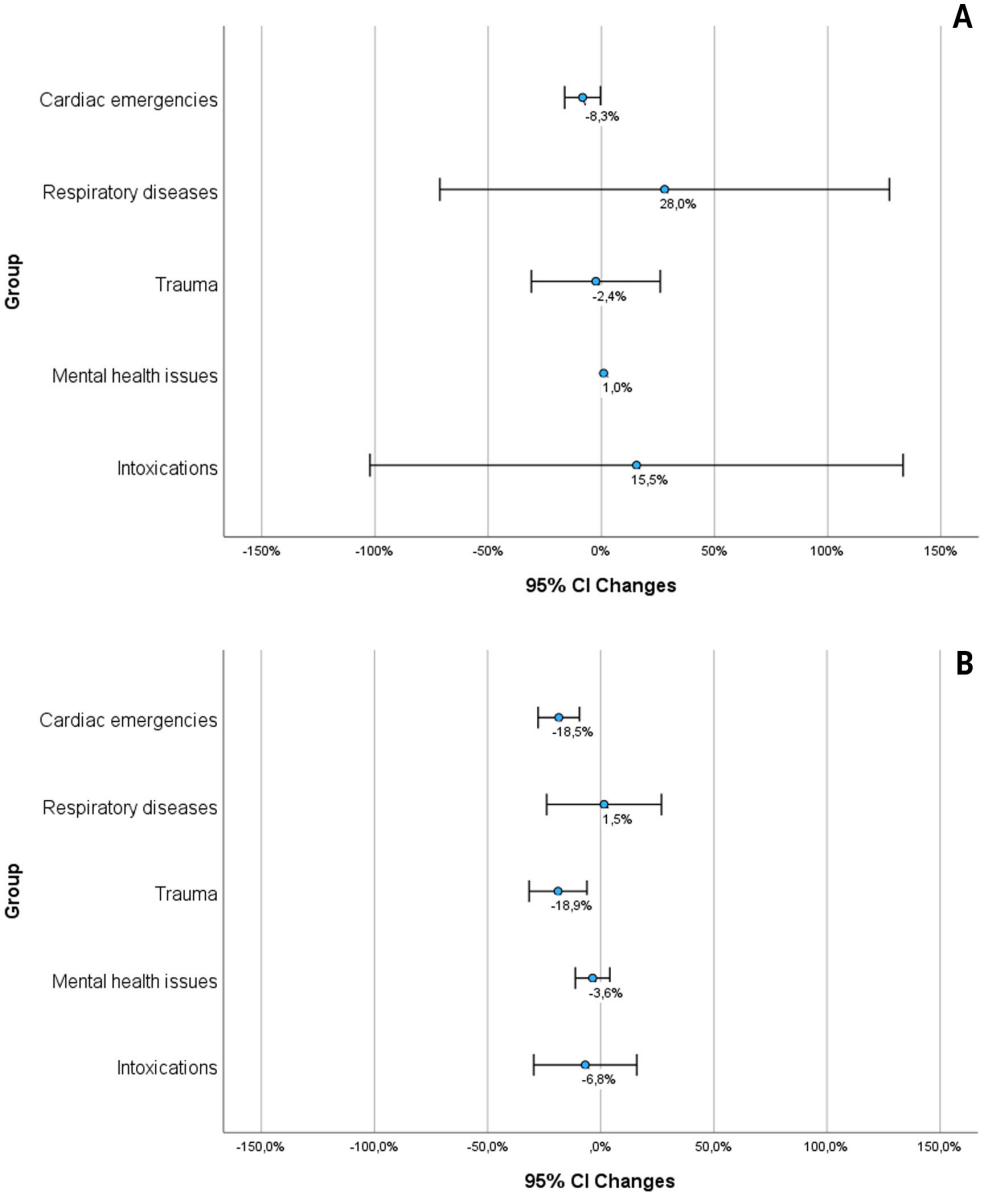
The figures show the graphical summary (mean and 95%-confidence interval) of the studies that reported quantitative data. **(A)** Shows the emergency calls, **(B)** shows the EMS operations, based on the sources: (16, 18, 20, 21, 37, 40, 44, 45, 49, 51, 54, 55, 57, 60, 63).

The articles that reported quantitative data on changes in EMS operations indicated an overall mean decrease of −12.2%, with a standard deviation of 24.7% (minimum: −72% to maximum: 56%) (Figure 5). Overall, 36 studies included changes in EMS operations (Supplementary material). Of these, 27 studies reported a decrease in EMS operations during the COVID-19 pandemic (15, 16, 19–21, 23, 36, 37, 39–41, 43–56, 58–60), varying from −2.6% (55) to −52.4% (16) (Supplementary material). Countries for which a decrease was reported were Japan (19, 36, 52), Portugal (16), Germany (39, 41, 50, 51, 58, 59), New Zealand (37), South Korea (20, 43), the Netherlands (21, 53), Austria (44), Canada (40), Poland (45), Finland (15, 47), the USA (48, 54, 56), Lithuania (49), France (60), Israel (55) and Spain (23). An increase in EMS operations was reported in six studies (14, 38, 46, 61–63), varying from +8.32% (14) to +51.5% (38) and representing the situation in Italy (38, 61), the USA (14), Canada (46), Iran (62) and Turkey (63). Two studies (Germany (18) and Italy (57) stated no changes in EMS operation frequencies. One study from the USA reported mixed trends depending on the study period.

### 3.5 Diagnosis-specific changes regarding EMS operations

EMS operations on cardiac emergencies, respiratory diseases, traumata, mental health conditions, and intoxication were considered (Supplementary material). Fourteen studies reported changes in EMS operations for cardiac emergencies (16, 18, 20, 21, 37, 40, 45, 49, 51, 54, 55, 57, 60, 63). One study reported an increase, nine reported a decrease, and three reported mixed trends depending on the specific diagnosis. In addition, one article reports no change. Nine studies examined changes in EMS operations for respiratory diseases (37, 38, 40, 44, 45, 50, 54, 55, 57). Six of them reported an increase, and three of them a decrease. Thirteen studies reported changes in traumata (15, 19, 23, 26, 37, 40, 44, 45, 48, 51, 54, 55, 57). One study stated an increase and ten studies stated a decrease of EMS operations for traumata. Two studies reported mixed trends depending on the specific diagnosis. Six studies reported changes in mental health conditions (37, 40, 45, 51, 54, 55); four reported an increase, and two a decrease. Five studies included changes in EMS operations for intoxication (37, 51, 54–56). Two described an increase, and three, a decrease in EMS operations.

## 4 Discussion

This review demonstrates the global impact of the coronavirus disease 2019 (COVID-19) pandemic on emergency medical services (EMS). We analyzed the number of emergency calls EMS operations and how these relate to specific diagnoses. Emergency calls showed no discernible trend, unlike in EMS operations, where frequencies decreased overall. This trend was also observed in the diagnoses of cardiac emergencies, traumata, and mental health conditions. On the other hand, respiratory diseases showed an increasing trend in EMS operations. EMS calls did not show a clear trend regarding specific diagnoses. However, considering the substantial data, heterogeneity results should be interpreted carefully. Data heterogeneity is caused by the pandemic's dynamic progression, resulting in variations in reported time intervals, study populations, and corresponding EMS systems.

While EMS operations decreased overall, studies from individual countries show increased EMS operations. So in Italy (+51.5% and +10%) (38, 61), an increase was reported from Iran (+21%) (62), an increase in Turkey (+9.8%) (63), an increase in California among older Latinos (+8.3%) (14), and an increase in Canada (+61%) (46). Apart from the different periods reported, it is challenging to identify geographical or structural correlations. A wide range of influences, such as the structure of the health care system, socio-demographic factors, and health policy decisions during the pandemic, need to be discussed.

The most significant effect, reported in almost all studies, might be caused by fear of getting infected with COVID-19 (14, 18, 21, 28, 51). This fear might have prevented people from seeking medical treatment (21, 27, 28, 61), for example, in hospitals (17–19, 21, 27, 29, 35, 40, 44, 45, 48, 51, 54–57, 59, 61). Aspects stimulating this fear were public information strategies (17, 55) with general recommendations to avoid hospitalization in case of non-emergency diseases (27, 47). However, it is also discussed that patients do not want to burden the healthcare system further (19, 21, 29, 40, 44, 46, 54), perhaps reinforced by media coverage from regions dramatically affected (40) and campaigns to thank healthcare providers (19). On the other hand, it is discussed that people were frightened by the media coverage, panicked, and called the emergency services to get information (63). Studies, when reported, classify differently from urgent or differently severely ill or injured patients. However, the proportion of non-life-threatening and, therefore, possibly “non-urgent bagatelle missions” has decreased by 58% in some cases (39).

The lockdown, with its social restrictions, also significantly impacted the number of EMS calls and operations. The closure of schools and workplaces, bars and nightclubs, and increased hygiene practices prevented many community-acquired illnesses (37). In addition, our scoping review demonstrates that patients with different diagnoses used the EMS more or less frequently during the COVID-19 pandemic. However, considering the results within the broader context of the general frequency of EMS calls and operations, along with their variations, is imperative. The periods examined in the individual studies included in this review were presented with significant heterogeneity. Nevertheless, when frequencies were previously available, the impression could be confirmed that the COVID-19 pandemic is the cause of the substantial fluctuations, as external literature also suggests (17, 37, 45, 47, 64).

### 4.1 Cardiac emergencies

Most of the studies included investigated cardiac emergencies in the broader sense during the COVID-19 pandemic. These included suspected diagnoses such as chest pain, myocardial infarction, cardiac arrest, stroke, and cerebral ischemia (Supplementary material). There is no clear trend in the emergency calls in the included studies, but the data indicate a decline in the number of emergency operations.

Changes in the utilization of EMS for cardiac emergencies and strokes have been described even in life-threatening health emergencies (18, 21, 22, 28, 33, 42, 46, 47, 49, 55, 63) leading to delayed response times or alerts to the emergency medical services (29, 42, 47, 55, 63). On the other hand, it was discussed whether the public recommendations on using emergency numbers led to increased EMS operations, particularly in the case of COVID-19 symptoms. The initial objective of this strategy was to prevent an undue burden on the emergency services, but it led to a delay in alerts and, therefore, in treatment. (20). However, it has been suggested that sudden professional and personal isolation also led to lifestyle changes (e.g., increased nicotine consumption and poorer medication adherence) in the home environment, which may be a further cause of the increase in cardiac emergencies and strokes (51). In contrast, decreased cardiovascular emergencies were explained by less intense physical activity, better air quality, and lower physiological and work-related stress in conjunction with COVID-19 measures, which could reduce the risk of acute myocardial infarction and stress-related cardiac events (21).

### 4.2 Respiratory diseases

COVID-19 is a systemic disease, so symptoms and the severity of a COVID-19 infection vary depending on the virus type. In addition to fever, respiratory symptoms such as respiratory distress and shortness of breath predominated. Therefore, many studies have examined the trends of EMS calls and operations for respiratory diseases, and there has been an increase. Increased operations caused by breathlessness were related to increased alertness of the population to these symptoms (14, 44). Reduced respiratory diseases have been linked to various factors like decreased social contact, decreased transmission of airborne illnesses (37), and improved air quality during the lockdown (37).

### 4.3 Trauma

Trauma-related incidents have declined sharply. There are many reasons for the decline in injuries and accidents, which are well explained. Traffic volumes fell sharply worldwide during the COVID-19 pandemic, accompanied by a significant decline in road deaths worldwide and reduced road fatalities (65). This is attributed to the general reduction in mobility, especially car traffic (23, 28, 37, 44, 45, 48, 52, 54, 55, 62), as the included studies also show, which is well explained by “stay at home” slogans (52, 54, 62, 63), the expansion of flexible working hours (63), home office jobs, and reduced commuter flow (51) l. However, road traffic accidents and trauma generally decreased (66, 67). Reasons for this decrease in EMS calls and operations due to traumata, in general, were related to the closure of nightclubs and bars (26, 37), an inability to socialize (37), canceled sports events and practices (26, 54), reduced risky recreational activities (48) and injury-prone locations (55).

### 4.4 Mental health conditions and intoxication

Studies examining EMS calls and operations due to mental health conditions showed a slight trend toward an increase. The increase in mental health conditions was associated with the restriction in social contacts and social isolation (37, 47), the fear of (1) Being infected with COVID-19 (37, 47), (2) losing family and friends through COVID-19 (37), and (3) losing one's job and therefore facing financial difficulties (37)—all possibly resulting in depression and anxiety (55). Psychosocial stress has also been discussed as a cause of an increase in domestic violence (23, 41, 51). Prison releases result in individuals abruptly reentering society, mainly without a care plan for people with opioid use disorder (56), and changes in the illicit drug market due to the social distancing measures (56). Other studies report an increase in psychosocial emergencies and higher suicide rates without providing a socio-spatial context (68, 69). A group that was reported to be severely affected by the restrictions due to COVID-19 were those using substances or those in recovery (28, 35, 42). Reasons discussed to explain this were social isolation, a lack of social support, stress, and particularly the interruption of (1) regular primary health care (28, 56), (2) medication supply for people with opioid use disorder, and (3) recovery support services (42). Concerning intoxication, no clear trend has been shown in the included studies.

### 4.5 Limitations

The principal challenge is the lack of comparability among the included studies due to differences in the reported study periods, the study populations and countries, and the corresponding EMS systems and contexts of comparison. Furthermore, the influence of the pandemic should be acknowledged not only in terms of emergency calls and operations but also in the treatment time for patients and the time required for ambulances to become operational due to hygiene protocols, which were not included in the review.

Since only articles in German and English were included, developments in other language countries could not be considered if studies were published in the national language. In addition, several other publications emerged during the research, but these were not included after the research period.

## 5 Conclusion

This scoping review shows that COVID-19 significantly impacted EMS calls and operations worldwide. While some studies report increased EMS calls and others decreased, a clear trend toward reduced EMS operations is evident. Specific patient groups used emergency services differently during the pandemic, influenced by social restrictions, lockdowns, and lifestyle changes. These findings highlight the need for improved preparedness for future crises.

Hospital studies reveal high workloads and increased mortality, yet a critical gap remains: EMS data are rarely linked to hospital outcomes, limiting insights into patient trajectories. A standardized “minimum emergency data set” or core data points should be established globally to improve emergency care and crisis response. This would enable better data interoperability within EMS and between EMS and hospitals, supporting more informed decision-making. Given global differences in healthcare systems and data protection laws, adaptable frameworks are essential to facilitate secure data exchange.

Despite these challenges, EMS demonstrated agility and adaptability. Although managing infectious patients is not routine for EMS, they quickly adjusted protocols, stocked protective equipment, and adapted faster than other healthcare providers.

However, the immense burden on EMS personnel must not be overlooked. Beyond traditional roles, they contributed significantly through COVID-19 testing, vaccine delivery, increased sanitation, extended response times, patient education, and covering for isolated colleagues. Their dedication ensured continuity of emergency care despite extreme strain.

For future health crises, enhancing data interoperability, strengthening EMS-hospital collaboration, and supporting EMS personnel are key to ensuring resilience, optimizing resources, and maintaining high-quality emergency care.

## Data Availability

The original contributions presented in the study are included in the article/Supplementary material, further inquiries can be directed to the corresponding author.
