# Supplementary material for: Impact of the COVID-19 pandemic on prehospital emergency medical service: a scoping review
Source: Front Public Health. 2025 Mar 19;13:1543150. doi: 10.3389/fpubh.2025.1543150 (PMC11962900; doi:10.3389/fpubh.2025.1543150)
Supplement: Supplementary file 1 [file Table_1.docx]

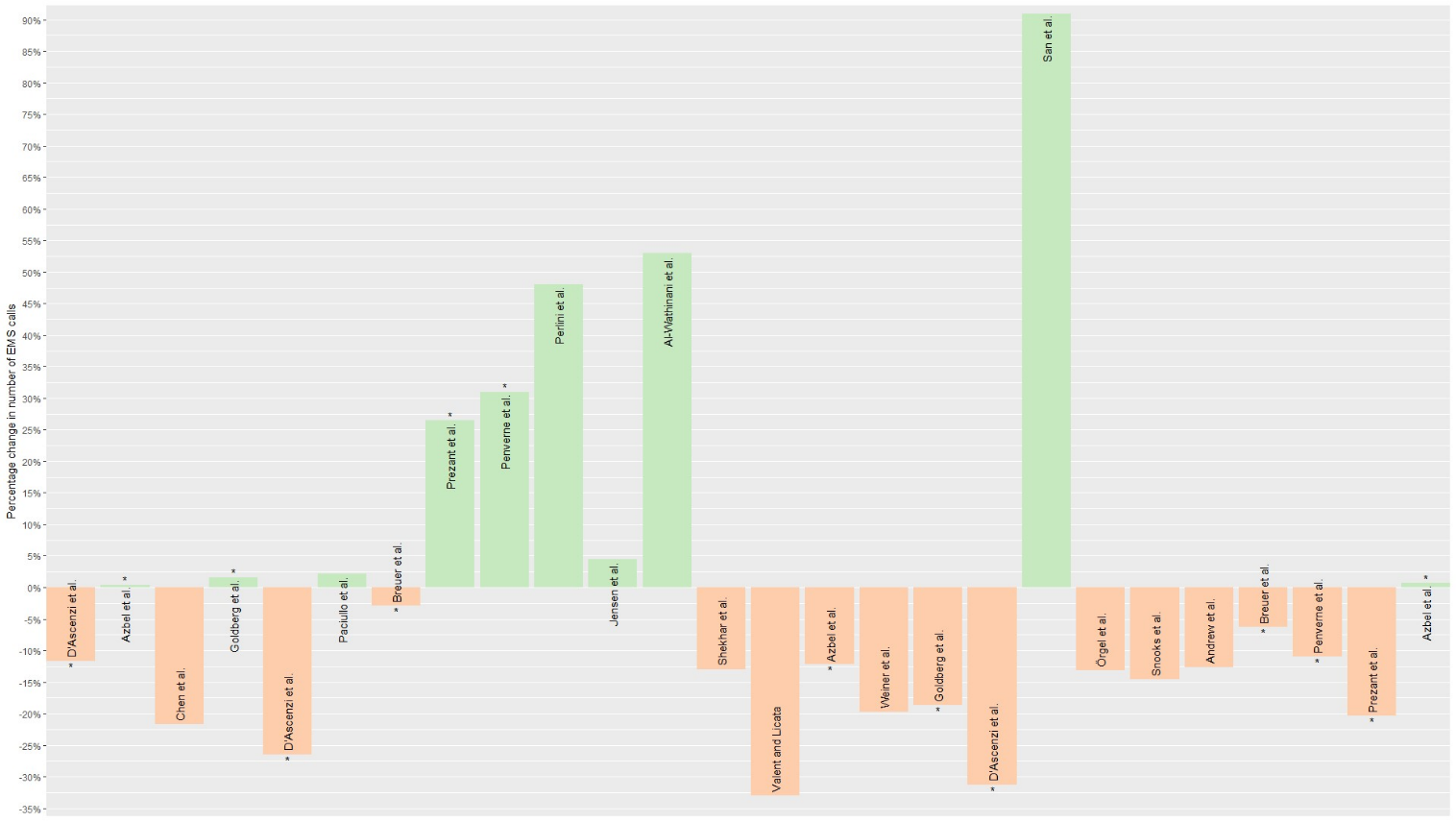


Studies' reported changes regarding EMS calls. In the graph, those studies that appear more than once (because they compared different periods) are marked with an asterisk (*)


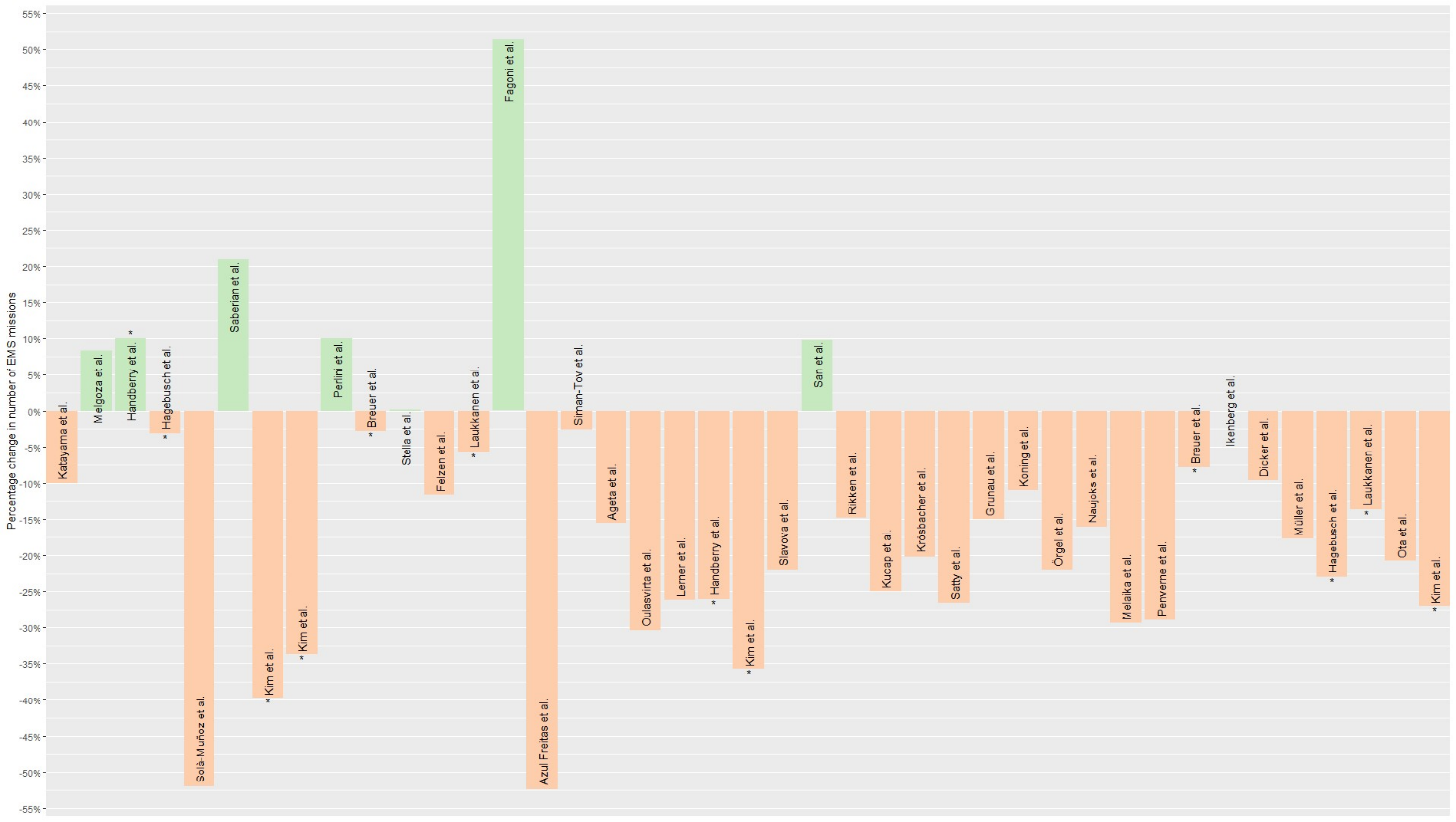


Studies' reported changes regarding EMS operations. In the graph, those studies that appear more than once (because they compared different periods) are marked with an asterisk (*)

**Table:** Studies' reported changes regarding EMS calls.

|  | Autor | Study periods | Changes* |
| --- | --- | --- | --- |
| Increase in EMS calls | Al-Wathinani et al. [24] | during covid-19 (03.20-04.20) vs. before covid-19 (01.20-02.20) | +52.95% |
|  | Jensen et al. [30] | during covid-19 (27.02.20-27.03.20) vs. corresponding period 2019 | +4.4% |
|  | Paciullo et al. [31] | during covid-19 (01.01.20-30.04.20) vs. corresponding period 2019 | +2.14% |
|  | Perlini et al. (Perlini et al., 2020) [61] | during covid-19 (21.02.20-26.03.20) vs. before covid-19 (17.01.20-20.02.20)  during covid-19 (21.02.20-26.03.20) vs. corresponding historical average data from the previous 5 years | +48%  +53% |
|  | Saberian et al. [62] | during covid-19 (18.02.20-16.03.20) vs. before covid-19 (21.01.20-17.02.20) | +347% |
|  | Şan et al. [63] | during covid-19 (11.03.20-24.04.20) vs. corresponding period 2018 and 2019 | +90.9% |
| Decrease in EMS calls | Andrew et al. [25] | lockdown (03.20 – 10.20) vs. before covid-19 (01.18 – 02.20) post-lockdown (11.20 – 02.21) vs. before covid-19 (01.18 – 02.20) | -12.6% -5.6% |
|  | Chen et al. [27] | during covid-19 (01.20-03.20) vs. corresponding period 2019 | -21,63% |
|  | D'Ascenzi et al. [17] | during covid-19 (01.01.-20.02.20) vs. corresponding period 2018 and 2019  during covid-19 (21.02.20-10.03.20) vs. corresponding period 2018 and 2019  lockdown (11.03.20-31.03.20) vs. corresponding period in 2018 and 2019 | -11.7%  -26.5%  -31.3% |
|  | Örgel et al. [59] | lockdown (16.03.20-16.04.20) vs. corresponding period 2018  lockdown (16.03.20-16.04.20) vs. corresponding period 2018 and 2019 | -17.4%  -13.1% |
|  | Shekhar et al. [22] | changes from 01.01.20-30.04.20  changes from 01.01.20-31.03.20  changes from 01.02.20-31.03.20  changes from 01.03.20-30.04.20 | -21.95%  -10.33%  -4.62%  -12.96% |
|  | Snooks et al. [33] | changes from 01.02.20-03.07.20 | -14.6% |
|  | Valent & Licata [34] | lockdown (03.-05.20) vs. corresponding period 2019  lockdown (03.-05.20) vs. corresponding period 2018 | -33.02%  -31.97% |
|  | Weiner et al. [35] | during covid-19 (10.03.20-15.05.20) vs. before covid-19 (15.02.20-09.03.20) | -19.7% |
|  | Ferron et al. [28] | 01.01.20-26.05.20 vs. corresponding period from 2016 to 2019 | decrease |
| Increase and Decrease in EMS calls | Azbel et al. [26] | pre-lockdown (01.20-08.03.20) vs. corresponding period in 2018 and 2019  lockdown (09.03.20-05.20) vs. corresponding period 2018 and 2019  post-lockdown (06.20-07.20) vs. corresponding period 2018 and 2019 | +0.4%  -12.2%  +0.7% |
|  | Breuer et al. [58] | during covid-19 (21.02.20-30.04.20) vs. year before (03.19)  during covid-19 (21.02.20-30.04.20) vs. before covid-19 (01.20)  lockdown (18.03.20-30.04.20) vs. pre-lockdown (21.02.20-18.03.20)  lockdown (18.03.20-30.04.20) vs. year before (03.19)  lockdown (18.03.20-30.04.20) vs. before covid-19 (01.20) | -2.9%  +1.44%  -8.85%  -6.25%  -2.06% |
|  | Goldberg et al. [29] | Pre-lockdown (15.02.20-10.03.20) vs. corresponding period 2019  lockdown (10.03.20-15.05.20) vs. corresponding period 2019 | +1.61%  -18.7% |
|  | Penverne et al. [60] | start of lockdown (17.03.20-29.03.20) vs. corresponding period 2019  start of lockdown (17.03.20-29.03.20) vs. corresponding period 2018  further course of lockdown (30.03.20-11.05.20) vs. corresponding period 2019  further course of lockdown (30.03.20-11.05.20) vs. corresponding period 2018 | +31%  +33%  -11%  -13% |
|  | Prezant et al. [32] | peak of covid-19 (16.03.20-15.04.20) vs. corresponding period 2019  post-surge period (16.04.20-31.05.20) vs. corresponding period 2019 | +26.45%  -20.37% |

*If no percentage change was presented in an article and no percentage change could be calculated, the table included whether an increase, a decrease, or no change was presented

**Table** Studies' reported diagnosis-specific changes regarding EMS calls

|  | Author | Diagnosis | Study periods | Changes* |
| --- | --- | --- | --- | --- |
| Increase in EMS calls for cardiac emergencies | Penverne et al. [60] | chestpain  cerebrovascular accident | lockdown (17.03.20-11.05.20) vs. corresponding period 2019  lockdown (17.03.20-11.05.20) vs. corresponding period 2019  lockdown (17.03.20-11.05.20) vs. corresponding period 2018 | +43%  +6.7%  +12% |
|  | Al-Wathinani et al. [24] | All cardiac diseases | during covid-19 (01.03.20-23.04.20) vs. before covid-19 (01.01.20-29.02.20) | +26.6% |
|  | Andrew et al. [25] | Acute coronary syndrome | lockdown (03.20 – 10.20) vs. before covid-19 (01.18 – 02.20) | increase |
|  | Prezant et al. [32] | Cardiovascular  Cardiopulmonary arrest  Other cardiac | peak of covid-19 (16.03.20-15.04.20) vs. corresponding period 2019 | increase  increase  increase |
|  | Valent & Licata [34] | Cardiac causes | lockdown (03.-05.20) vs. corresponding period 2019  lockdown (03.-05.20) vs. corresponding period 2018 | increase  increase |
| Decrease in EMS calls for cardiac emergencies | Chen et al. [27] | Cardiovascular diseases | during covid-19 (01.20-03.20) vs. corresponding period 2019 | -32.86% |
|  | D'Ascenzi et al. [17] | All cardiac diseases | during covid-19 (01.01.-20.02.20) vs. corresponding period 2018 and 2019  during covid-19 (21.02.20-10.03.20) vs. corresponding period 2018 and 2019  lockdown (11.03.20-31.03.20) vs. corresponding period in 2018 and 2019 | -11.7%  -26.5%  -31.3% |
|  | Paciullo et al. [31] | ACS  Stroke and cardiac arrest | during covid-19 (01.01.20-30.04.20) vs. corresponding period 2019 | decrease  decrease |
| Increase and Decrease in EMS calls for cardiac emergencies | Ferron et al. [28] | Cardiac arrest  Chest pain  Heart problems  Stroke | 01.01.20-26.05.20 vs. corresponding period from 2016 to 2019 | +26%  -3%  +17%  +13% |
|  | Shekhar et al. [22] | All cardiac diseases  STEMI  Stroke alert  Asystole | changes from 01.01.20-30.04.20  changes from 01.01.20-31.03.20  changes from 01.02.20-31.03.20  changes from 01.03.20-30.04.20  changes from 01.01.20-30.04.20  changes from 01.01.20-31.03.20  changes from 01.02.20-31.03.20  changes from 01.03.20-30.04.20  changes from 01.01.20-30.04.20  changes from 01.01.20-31.03.20  changes from 01.02.20-31.03.20  changes from 01.03.20-30.04.20  changes from 01.01.20-30.04.20  changes from 01.01.20-31.03.20  changes from 01.02.20-31.03.20  changes from 01.03.20-30.04.20 | -30.07%  -18%  -12.8%  -14.66%  -18.71%  -15.4%  -17.37%  -3.91%  -16.60%  -10.95%  -7.56%  -6.34%  +14.46%  -10.11%  -5.36%  +27.34% |
|  | Goldberg et al. [29] | Cardiac emergencies  Stroke  Cardiac arrest | Post-covid-19 (10.03.20-15.05.20) vs. corresponding period 2019  Post-covid-19 (10.03.20-15.05.20) vs. corresponding period 2019  Post-covid-19 (10.03.20-15.05.20) vs. corresponding period 2019 | -35.6%  -12.3%  +18.2% |
| Increase in EMS calls for respiratory diseases | Penverne et al. [60] | Breathing difficulties | lockdown (17.03.20-11.05.20) vs. corresponding period 2019 | +121% |
|  | Goldberg et al. [29] | Respiratory problems | Post-covid-19 (10.03.20-15.05.20) vs. corresponding period 2019 | +4.5% |
|  | Prezant et al. [32] | Respiratory problems  Asthma | peak of covid-19 (16.03.20-15.04.20) vs. corresponding period 2019 | increase  increase |
|  | Valent & Licata [34] | Respiratory causes | lockdown (03.-05.20) vs. corresponding period 2019  lockdown (03.-05.20) vs. corresponding period 2018 | increase  increase |
| Decrease in EMS calls for respiratory diseases | Ferron et al. [28] | Breathing problems | 01.01.20-26.05.20 vs. corresponding period from 2016 to 2019 | -12% |
|  | Weiner et al. [35] | Respiratory complaints | during covid-19 (10.03.20-15.05.20) vs. before covid-19 (15.02.20-09.03.20) | -1.7% |
| Decrease in EMS calls for traumata | Penverne et al. [60] | Severe trauma  Minor injuries | lockdown (17.03.20-11.05.20) vs. corresponding period 2019  lockdown (17.03.20-11.05.20) vs. corresponding period 2019 | -50%  -22% |
|  | Azbel et al. [26] | All traumas | lockdown (09.03.20-05.20) vs. corresponding period 2018 and 2019 | -23.3% |
|  | Al-Wathinani et al. [24] | All traumas | during covid-19 (01.03.20-23.04.20) vs. before covid-19 (01.01.20-29.02.20) | -6.1% |
|  | Prezant et al. [32] | Blunt trauma  Other trauma | peak of covid-19 (16.03.20-15.04.20) vs. corresponding period 2019 | decrease  decrease |
|  | Valent & Licata [34] | Trauma | lockdown (03.-05.20) vs. corresponding period 2019  lockdown (03.-05.20) vs. corresponding period 2018 | decrease  decrease |
| Increase and Decrease in EMS calls for traumata | Ferron et al. [28] | Drowning/Diving accident  Falls  Inaccessible accident  Stab/Gunshot  Motor vehicle collision  Traumatic injuries | 01.01.20-26.05.20 vs. corresponding period from 2016 to 2019 | -23%  +2%  +90%  +40%  -17%  -15% |
| Increase in EMS calls for mental health conditions | Ferron et al. [28] | Psychiatric/Abnormal Behaviour | 01.01.20-26.05.20 vs. corresponding period from 2016 to 2019 | +1% |
|  | Andrew et al. [25] | Mental health issues | lockdown (03.20 – 10.20) vs. before covid-19 (01.18 – 02.20) | increase |
| Decrease in EMS calls for mental health conditions | Prezant et al. [32] | Psych/drug | peak of covid-19 (16.03.20-15.04.20) vs. corresponding period 2019 | decrease |
| Increase in EMS calls for intoxication | Ferron et al. [28] | intoxication | 01.01.20-26.05.20 vs. corresponding period from 2016 to 2019 | +70% |
| Decrease in EMS calls for intoxication | Weiner et al. [35] | substance-related issues | during covid-19 (10.03.20-15.05.20) vs. before covid-19 (15.02.20-09.03.20) | -16.4% |
|  | Penverne et al. [60] | Intoxication | lockdown (17.03.20-11.05.20) vs. corresponding period 2019 | -7% |

*If no percentage change was presented in an article and no percentage change could be calculated, the table included whether an increase, a decrease, or no change was presented

**Table** Studies' reported changes regarding EMS operations.

|  | Autor | Study periods | Changes* |
| --- | --- | --- | --- |
| Increase in EMS operations | Fagoni et al. [38] | peak of covid-19 (03.20-04.20) vs. corresponding period 2019 | +51.5% |
|  | Melgoza et al. [14] | during covid-19 (01.20-06.20) vs. corresponding period 2019 | +8.32% |
|  | Perlini et al. [61] | during covid-19 (21.02.20-26.03.20) vs. before covid-19 (17.01.20-20.02.20)  during covid-19 (21.02.20-26.03.20) vs. corresponding historical average data from the previous 5 years | +10%  +22% |
|  | Saberian et al. [62] | during covid-19 (18.02.20-16.03.20) vs. before covid-19 (21.01.20-17.02.20) | +21% |
|  | Şan et al. [63] | during covid-19 (11.03.20-24.04.20) vs. corresponding period 2018 and 2019 | +9.8% |
|  | Lane et al. [46] | during covid-19 (12.19-06.20) vs. corresponding period 2017-2018 and 2018-2019 | increase |
| Decrease in EMS operations | Ageta et al. [36] | during covid-19 (03.20-05.20) vs. corresponding period 2019  during covid-19 (03.20) vs. corresponding period 2019  during covid-19 (04.20) vs. corresponding period 2019  during covid-19 (05.20) vs. corresponding period 2019 | -15.5%  -7.8%  -16.8%  -21.8% |
|  | Azul Freitas et al. [16] | during covid-19 (03.20-04.20) vs. corresponding period 2019 | -52.4% |
|  | Breuer et al. [58] | during covid-19 (21.02.20-30.04.20) vs. year before (03.19)  during covid-19 (21.02.20-30.04.20) vs. before covid-19 (01.20)  lockdown (18.03.20-30.04.20) vs. pre-lockdown (21.02.20-18.03.20)  lockdown (18.03.20-30.04.20) vs. year before (03.19)  lockdown (18.03.20-30.04.20) vs. before covid-19 (01.20) | -2.83%  -5.58%  -12.69%  -7.81%  -10.42% |
|  | Dicker et al. [37] | lockdown (23.03.20-26.04.20) vs. pre-lockdown (01.03.18-30.11.18 and 01.07.19-16.02.20) | -9.6% |
|  | Felzen et al. [39] | during covid-19 (03.20) vs. corresponding period 2019  during covid-19 (03.20) vs. corresponding period 2018 | -11.57%  -15.56% |
|  | Grunau et al. [40] | during covid-19 (15.03.20-15.05.20) vs. corresponding period 2019 | -15% |
|  | Hagebusch et al. [41] | lockdown (23.03.20-03.05.20) vs. the mean of 2018 and 2019  pre-lockdown (10.02.20-22.03.20) vs. the mean of 2018 and 2019 | -23.02%  -3.1% |
|  | Katayama et al. [19] | during covid-19 (01.01.20-14.04.20) vs. corresponding period 2019 | -10.01% |
|  | Kim et al. [43] | during covid-19 (19.02.20-03.03.20) vs. before covid-19 (01.01.-18.02.)  during covid-19 (04.03.20-31.03.20) vs. before covid-19 (01.01.-18.02.)  during covid-19 (01.04.20-30.04.20) vs. before covid-19 (01.01.-18.02.) | -39.7%  -35.7%  -27% |
|  | Kim et al. [20] | during covid-19 (19.02.20-20.04.20) vs. before covid-19 (20.11.19-20.01.20) | -33.7% |
|  | Koning et al. [21] | lockdown (16.03.20-26.04.20) vs. corresponding period 2019 | -11% |
|  | Krösbacher et al. [44] | peak of covid-19 (15.03.20-15.05.20) vs. corresponding period 2017-2019 | -20.2% |
|  | Kucap et al. [45] | during covid-19 (15.03.20-13.05.20) vs. corresponding period 2018 and 2019 | -25% |
|  |  |  |  |
|  | Laukkanen et al. [47] | during covid-19 (03.20) vs. corresponding period 2016-2019  during covid-19 (04.20) vs. corresponding period 2016-2019 | -5.7%  -13.6% |
|  | Lerner et al. [48] | changes from 02.03.20-08.03.20 to 13.04.20-19.04.20 | -26.1% |
|  | Melaika et al. [49] | lockdown (16.03.20-16.06.20) vs. pre-lockdown (01.12.19-15.03.20) | -29.41% |
|  | Müller et al. [50] | lockdown (23.03.20-03.05.20) vs. pre-lockdown (10.02.20-22.03.20) | -17.7% |
|  | Naujoks et al. [51] | lockdown (16.03.20 – 24.05.20) vs. pre-lockdown (06.01.20-15.03.20) | -16% |
|  | Örgel et al. [59] | lockdown (16.03.20-16.04.20) vs. corresponding period 2018 and 2019 | -22% |
|  | Ota et al. [52] | during covid-19 (04.20-03.21) vs. corresponding period 2019-2020 | -20.8% |
|  | Oulasvirta et al. [15] | during covid-19 (01.03.20-31.05.20) vs. corresponding period 2017-2019 | -30.4% |
|  | Penverne et al. [60] | lockdown (17.03.20-11.05.20) vs. corresponding period 2019 | -29% |
|  | Rikken et al. [53] | lockdown (13.03.20-13.05.20) vs. corresponding period 2019 | -14.8% |
|  | Satty et al. [54] | during covid-19 (15.03.20-15.05.20) vs. corresponding period 2016-2019 | -26.5% |
|  | Siman-Tov et al. [55] | during covid-19 (03.20-04.20) vs. corresponding period 2019 | -2.6% |
|  | Slavova et al. [56] | during covid-19 (06.03.20-26.04.20) vs. before covid-19 (14.01.20-05.03.20) | -22% |
|  | Solà-Muñoz et al. [23] | during covid-19 (15.02.20-15.05.20) vs. corresponding period 2019 | -52% |
| Increase and Decrease in EMS operations | Handberry et al. [42] | 2020 vs. 2019  changes from 02.03.20-19.04.20 | +10%  -26% |
| No change in EMS operations | Stella et al. [57] | during covid-19 (21.02.20 – 03.04.20) vs. before covid-19 (10.01.20-20.02.20) | +0.2% |
|  | Ikenberg et al. [18] | lockdown (21.03.20-19.04.20) vs. pre-lockdown (01.01.20-20.03.20) | No change |

*If no percentage change was presented in an article and no percentage change could be calculated, the table included whether an increase, a decrease, or no change was presented

**Table** Studies' reported diagnosis-specific changes regarding EMS operations

|  | Autor | Diagnosis | Study periods | Changes* |
| --- | --- | --- | --- | --- |
| Increase in EMS operations for cardiac emergencies | Dicker et al. [37] | All cardiac diseases  Stroke | lockdown (23.03.20-26.04.20) vs. pre-lockdown (01.03.18-30.11.18 and 01.07.19-16.02.20) | +1.0%  +0.2% |
| Decrease in EMS operations for cardiac emergencies | Azul Freitas et al. [16] | STEMI | during covid-19 (03.20-04.20) vs. corresponding period 2019 | -52.4% |
|  | Stella et al. [57] | out-of-hospital cardiac arrests | during covid-19 (21.02.20 – 03.04.20) and before covid-19 (10.01.20-20.02.20) vs. corresponding periods 2019 | -38% |
|  | Şan et al. [63] | Angina pectoris  Myocardial infarction | during covid-19 (11.03.20-24.04.20) vs. corresponding period 2019  during covid-19 (11.03.20-24.04.20) vs. corresponding period 2019 | -35.2%  -45% |
|  | Kim et al. [20] | Stroke | during covid-19 (19.02.20-20.04.20) vs. before covid-19 (20.11.19-20.01.20) | -33.7% |
|  | Naujoks et al. [51] | Cerebral ischemia  Cardiac ischemia | lockdown (16.03.20 – 24.05.20) vs. pre-lockdown (06.01.20-15.03.20)  lockdown (16.03.20 – 24.05.20) vs. pre-lockdown (06.01.20-15.03.20) | -20%  -20% |
|  | Penverne et al. [60] | STEMI | lockdown (17.03.20-11.05.20) vs. corresponding period 2019 | -12% |
|  | Melaika et al. [49] | Stroke | lockdown (16.03.20-16.06.20) vs. pre-lockdown (01.12.19-15.03.20) | decrease |
|  | Koning et al. [21] | Chest pain  STEMI | lockdown (16.03.20-26.04.20) vs. corresponding period 2019 | decrease  decrease |
|  | Grunau et al. [40] | Chest pain  Stroke  STEMI | during covid-19 (15.03.20-15.05.20) vs. corresponding period 2019 | decrease  decrease  decrease |
| Increase and Decrease in EMS operations for cardiac emergencies | Siman-Tov et al. [55] | Cardiovascular diseases  Cardiac arrest | during covid-19 (03.20-04.20) vs. corresponding period 2019  during covid-19 (03.20-04.20) vs. corresponding period 2019 | -8.01%  +15.82% |
|  | Kucap et al. [45] | Sudden cardiac arrest  Chestpain  Cardiological problems | during covid-19 (15.03.20-13.05.20) vs. corresponding period 2018  during covid-19 (15.03.20-13.05.20) vs. corresponding period 2019  during covid-19 (15.03.20-13.05.20) vs. corresponding period 2018  during covid-19 (15.03.20-13.05.20) vs. corresponding period 2019  during covid-19 (15.03.20-13.05.20) vs. corresponding period 2018  during covid-19 (15.03.20-13.05.20) vs. corresponding period 2019 | +12.52%  +6.36%  -17.22%  -16.1%  -4.49%  -15.27% |
| No change in EMS operations for cardiac emergencies | Ikenberg et al. [18] | Stroke | lockdown (21.03.20-19.04.20) vs. pre-lockdown (01.01.20-20.03.20) | No change |
| Increase and no change in EMS operations for cardiac emergencies | Satty et al. [54] | Cardiac arrest  Cardiac disease  stroke | during covid-19 (15.03.20-15.05.20) vs. corresponding period 2016-2019 | +0.8%  No change  +0.2% |
| Increase in EMS operations for respiratory diseases | Stella et al. [57] | Respiratory distresses | during covid-19 (21.02.20 – 03.04.20) and before covid-19 (10.01.20-20.02.20) vs. corresponding periods 2019 | +56% |
|  | Siman-Tov et al. [55] | Respiratory diseases | during covid-19 (03.20-04.20) vs. corresponding period 2019 | +20.8% |
|  | Krösbacher et al. [44] | shortness of breath | peak of covid-19 (15.03.20-15.05.20) vs. corresponding period 2017-2019 | +18.7% |
|  | Satty et al. [54] | Respiratory diseases | during covid-19 (15.03.20-15.05.20) vs. corresponding period 2016-2019 | +0.6% |
|  | Fagoni et al. [38] | Breathing problems | peak of covid-19 (03.20-04.20) vs. corresponding period 2019 | increase |
|  | Grunau et al. [40] | Respiratory distress/ symptoms | during covid-19 (15.03.20-15.05.20) vs. corresponding period 2019 | increase |
| Decrease in EMS operations for respiratory diseases | Müller et al. [50] | Respiratory problems | lockdown (23.03.20-03.05.20) vs. pre-lockdown (10.02.20-22.03.20) | -40.6% |
|  | Kucap et al. [45] | Dyspnoea | during covid-19 (15.03.20-13.05.20) vs. corresponding period 2018  during covid-19 (15.03.20-13.05.20) vs. corresponding period 2019 | -23.73%  -18.14% |
|  | Dicker et al. [37] | Respiratory problems | lockdown (23.03.20-26.04.20) vs. pre-lockdown (01.03.18-30.11.18 and 01.07.19-16.02.20) | -1.8% |
| Increase in EMS operations for traumata | Oulasvirta et al. [15] | Trauma | during covid-19 (01.03.20-31.05.20) vs. corresponding period 2017-2019 | +23.7% |
| Decrease in EMS operations for traumata | Stella et al. [57] | Major trauma | during covid-19 (21.02.20 – 03.04.20) and before covid-19 (10.01.20-20.02.20) vs. corresponding periods 2019 | -72% |
|  | Kucap et al. [45] | Traffic accident  Injuries | during covid-19 (15.03.20-13.05.20) vs. corresponding period 2018  during covid-19 (15.03.20-13.05.20) vs. corresponding period 2019  during covid-19 (15.03.20-13.05.20) vs. corresponding period 2018  during covid-19 (15.03.20-13.05.20) vs. corresponding period 2019 | -53.48%  -51.17%  -38.52%  -32.3% |
|  | Azbel et al. [26] | Injuries due to alcohol intoxication | lockdown (09.03.20-05.20) vs. corresponding period 2018 and 2019 | -41.8% |
|  | Naujoks et al. [51] | Polytrauma | lockdown (16.03.20 – 24.05.20) vs. pre-lockdown (06.01.20-15.03.20) | -34% |
|  | Krösbacher et al. [44] | Traffic accidents | peak of covid-19 (15.03.20-15.05.20) vs. corresponding period 2017-2019 | -26.4% |
|  | Siman-Tov et al. [55] | Traumatic injuries | during covid-19 (03.20-04.20) vs. corresponding period 2019 | -18.87% |
|  | Katayama et al. [19] | Traffic accidents | during covid-19 (01.01.20-14.04.20) vs. corresponding period 2019 | -8.4% |
|  | Satty et al. | Trauma | during covid-19 (15.03.20-15.05.20) vs. corresponding period 2016-2019 | -1.1% |
|  | Grunau et al. [40] | Trauma | during covid-19 (15.03.20-15.05.20) vs. corresponding period 2019 | decrease |
|  | Lerner et al. [48] | Potential injuries | changes from 02.03.20-08.03.20 to 13.04.20-19.04.20 and the same time period in previous years | decrease |
| Increase and Decrease in EMS operations for traumata | Solà-Muñoz et al. [23] | Road traffic accidents  pedestrian-vehicle collisions  injuries caused by weapons  burns | during covid-19 (15.02.20-15.05.20) vs. corresponding period 2019 | -56.9%  -63%  -4.65%  +46.7% |
|  | Dicker et al. [37] | All traumata  non-alcohol related traumata  alcohol related traumata  Assault-related injuries  Fall  Other traumata  Road traffic crash | lockdown (23.03.20-26.04.20) vs. pre-lockdown (01.03.18-30.11.18 and 01.07.19-16.02.20) | -1.7%  +1.9%  -1.9%  -0.3%  +8.0%  +2.3%  -10.0% |
| Increase in EMS operations for mental health conditions | Siman-Tov et al. [55] | Psychiatry | during covid-19 (03.20-04.20) vs. corresponding period 2019 | +4.26% |
|  | Dicker et al. [37] | Mental health issues | lockdown (23.03.20-26.04.20) vs. pre-lockdown (01.03.18-30.11.18 and 01.07.19-16.02.20) | +1.3% |
|  | Satty et al. [54] | Psychiatric/Behavioral | during covid-19 (15.03.20-15.05.20) vs. corresponding period 2016-2019 | +0.3% |
|  | Grunau et al. [40] | Anxiety | during covid-19 (15.03.20-15.05.20) vs. corresponding period 2019 | increase |
| Decrease in EMS operations for mental health conditions | Naujoks et al. [51] | Psychiatric emergencies | lockdown (16.03.20 – 24.05.20) vs. pre-lockdown (06.01.20-15.03.20) | -16% |
|  | Kucap et al. [45] | Mental disorders | during covid-19 (15.03.20-13.05.20) vs. corresponding period 2018  during covid-19 (15.03.20-13.05.20) vs. corresponding period 2019 | -5.3%  -6.1% |
| Increase in EMS operations for intoxication | Slavova et al. [56] | Opioid overdose | during covid-19 (06.03.20-26.04.20) vs. before covid-19 (14.01.20-05.03.20) | +17% |
|  | Satty et al. [54] | Toxicological | during covid-19 (15.03.20-15.05.20) vs. corresponding period 2016-2019 | +0.4% |
| Decrease in EMS operations for intoxication | Naujoks et al. [51] | Intoxications | lockdown (16.03.20 – 24.05.20) vs. pre-lockdown (06.01.20-15.03.20) | -27% |
|  | Siman-Tov et al. [55] | Overdose | during covid-19 (03.20-04.20) vs. corresponding period 2019 | -23.65% |
|  | Dicker et al. [37] | Poisoning | lockdown (23.03.20-26.04.20) vs. pre-lockdown (01.03.18-30.11.18 and 01.07.19-16.02.20) | -0.9% |

*If no percentage change was presented in an article and no percentage change could be calculated, the table included whether an increase, a decrease, or no change was presented
